# Chinese college teachers’ emotional intelligence and mental health: a chain mediation model involving student relationship quality

**DOI:** 10.3389/fpsyg.2025.1572070

**Published:** 2025-07-24

**Authors:** Qian Zhang

**Affiliations:** School of International Culture, South China Normal University, Guangzhou, China

**Keywords:** teacher engagement, mental health, emotional intelligence, teacher–student relationships, Chinese college teachers

## Abstract

**Introduction:**

This study investigates the relationship between teacher engagement and mental health among Chinese college teachers, examining the mediating roles of emotional intelligence (EI) and teacher-student relationships. Teacher engagement, characterized by commitment, enthusiasm, and dedication, is crucial for educators’ mental well-being, as it fosters job satisfaction, reduces stress, and mitigates burnout. In the context of Chinese higher education, unique challenges such as heavy workloads and high expectations necessitate targeted approaches to enhance teacher engagement and mental health.

**Methods:**

The study utilized a four-wave panel design involving 1,264 Chinese college teachers from Guangdong Province. Data were collected on teacher engagement, EI components (attention, clarity, and repair), the quality of teacher-student relationships, and mental health outcomes.

**Results:**

The results revealed a significant direct effect of teacher engagement on mental health, indicating that higher engagement levels are associated with better mental health outcomes. Mediation analyses showed that EI attention and repair significantly mediated this relationship, while EI clarity did not independently mediate the effect. Additionally, serial mediation analysis highlighted the importance of EI clarity and teacher-student relationships in enhancing mental health.

**Discussion:**

Engaged teachers with high EI were better equipped to manage stress and build positive relationships with their students, fostering a supportive work environment. The study underscores the need for professional development programs focusing on EI training and strategies to improve teacher-student relationships, aiming to support teachers’ well-being. Overall, this research provides valuable insights into the complex interplay between teacher engagement, emotional intelligence, and mental health, offering guidance for educational institutions to develop targeted interventions that promote teacher well-being and effectiveness.

## Introduction

Teacher mental health is a critical aspect of the educational environment, significantly impacting both educators and students. Mental well-being among teachers is essential for maintaining high levels of job performance, fostering positive teacher–student relationships, and creating a supportive learning environment. In the context of Chinese college education, teachers face unique pressures and demands, such as heavy workloads, high expectations, and potential isolation. These challenges necessitate a focused approach to promote the mental health and engagement of educators (Wang, 2024).

The reform of college teaching in Guangdong province underscores the urgent need to address the marginalization of Chinese education among students and teachers. By implementing innovative teaching systems and enhancing the quality of educators, the status and effectiveness of Chinese education can be improved (Chen, 2017). Such reforms can foster a more supportive and engaging environment for teachers, contributing significantly to their mental well-being.

Understanding the mental health of teachers is pivotal as it influences their ability to engage effectively with their students and perform their duties efficiently. Teacher engagement, defined by commitment, enthusiasm, and dedication to teaching, has been identified as a crucial factor influencing the mental well-being of educators (Xu et al., 2023). High levels of engagement are linked to greater job satisfaction, which is essential for positive mental health outcomes (Mazzetti et al., 2023). Engaged teachers find meaning and fulfillment in their work, leading to reduced stress and a lower risk of burnout (Prasojo et al., 2020).

Furthermore, teacher engagement contributes to the creation of positive work environments that foster supportive and collaborative professional communities. These environments benefit students’ experiences and offer emotional support to teachers, reducing feelings of isolation and stress (Maricuțoiu et al., 2023). The enthusiasm and commitment of engaged teachers enhance their ability to cope with work-related stress, serving as buffers against its detrimental effects (Ayyashi et al., 2024).

Professional growth and development are often linked with engagement, as these teachers actively seek opportunities to refine their skills and knowledge (Dai and Wang, 2023). This continuous development builds a sense of competence and efficacy, which helps reduce anxiety and depression (Holmstrom et al., 2023). Additionally, engaged teachers maintain a healthy work-life balance, deriving satisfaction from their work while preventing it from encroaching on their personal lives (Dudasova et al., 2024). This balance is crucial for overall well-being. Furthermore, engaged teachers show increased resilience and adaptability to changes and challenges within the educational landscape, effectively navigating difficulties to protect their mental health (Deroncele-Acosta et al., 2024).

Despite the established link between teacher engagement and positive mental health outcomes, understanding this relationship within the context of Chinese college teachers requires further exploration. Research highlights significant differences in the curricula of Chinese language teacher education programs between China and Australia, emphasizing the urgent need for an internationalized curriculum to meet the demand for qualified teachers (Wang et al., 2013). Addressing perceptual gaps in instructor immediacy compared to American counterparts can also improve teacher engagement and mental health (Myers et al., 1998). Targeted professional development and support are essential to bridge these gaps and promote engagement and well-being.

Emotional intelligence (EI) is increasingly recognized as a critical factor in mental health and workplace well-being (Salovey et al., 2002; Sha et al., 2022). EI includes the ability to perceive, understand, use, and manage emotions effectively (Seal et al., 2009). This study focuses on three components of EI: attention, clarity, and repair. Attention involves being aware of emotions, clarity entails understanding and identifying emotions accurately, and repair refers to effectively regulating and managing emotions.

Among these components, emotional clarity and repair are particularly relevant to the quality of interpersonal relationships in educational contexts. Emotional clarity allows teachers to make sense of their affective experiences, differentiate among emotional states, and recognize the antecedents of their emotions. This capacity enables them to respond thoughtfully in relationally demanding situations, such as conflict with students or emotionally charged classroom interactions. Clarity facilitates a more accurate interpretation of others’ emotions as well, which is foundational to empathy and mutual understanding—cornerstones of high-quality teacher–student relationships (Valente and Lourenço, 2022).

Similarly, emotional repair plays a central role in helping teachers regulate negative emotions and restore emotional balance following stressful encounters. Teachers with strong repair abilities are more likely to use adaptive coping strategies, such as reappraisal, self-soothing, or seeking social support, thereby reducing emotional exhaustion and enhancing relational resilience. This emotional recovery not only protects their own mental health but also allows them to maintain warmth, patience, and responsiveness in their interactions with students (Menter, 2022).

While previous studies have explored EI’s mediating role in general work settings, its specific impact on Chinese college teachers remains under-explored. Given the unique stressors faced by these educators, understanding EI’s influence on their engagement and mental health is crucial for developing targeted interventions. This study aims to fill this gap by examining the mediating role of EI—specifically attention, clarity, and repair—in the relationship between teacher engagement and mental health among Chinese college teachers. This approach seeks to provide insights into enhancing teacher well-being through the development of emotional intelligence skills.

Furthermore, exploring the serial mediation model between teacher engagement, EI, and teacher–student relationships on teachers’ mental health highlights the interconnectedness of these factors. Engaged teachers with high EI are better able to handle stress and build positive relationships with their students, fostering a supportive and fulfilling work environment (Bechter et al., 2023). Improved teacher–student relationships contribute to teachers’ mental health by providing emotional support and reducing feelings of isolation (Gan, 2021). This positive feedback loop, where better mental health further enhances engagement and EI, underscores the importance of comprehensive strategies to support teachers’ well-being. Understanding these intricate relationships is essential for developing targeted interventions to improve teachers’ effectiveness and well-being in educational settings.

## Literature review

### Chinese college teachers’ engagement and mental health

Teacher engagement, characterized by commitment, enthusiasm, and dedication to teaching, is emerging as a significant factor influencing the mental well-being of educators (Xu et al., 2023). Previous research suggests a strong correlation between heightened engagement and increased job satisfaction, a pivotal element in maintaining positive mental health (Mazzetti et al., 2023). Engaged teachers typically derive meaning and fulfillment from their work, leading to reduced stress and mitigating burnout risk (Prasojo et al., 2020).

Furthermore, engaged teachers contribute to the creation of positive work environments, promoting supportive and collaborative professional communities, which ultimately impact positively on students’ experiences (Maricuțoiu et al., 2023). Such environments offer emotional support and reduce feelings of isolation and stress, which are known to negatively impact mental health. The inherent enthusiasm and commitment of engaged teachers also enhance coping mechanisms for dealing with work-related stress, serving as buffers against its detrimental effects (Ayyashi et al., 2024).

Professional growth and development are often synonymous with engagement, as engaged teachers actively seek opportunities to refine their skills and knowledge (Dai and Wang, 2023). This ongoing development fosters a sense of competence and efficacy, reducing anxiety and depression levels (Holmstrom et al., 2023). Maintaining a healthy work-life balance is another hallmark of engaged teachers, who derive satisfaction from their work, preventing it from encroaching on their personal lives (Dudasova et al., 2024). This equilibrium is crucial for overall well-being.

Additionally, engaged teachers exhibit increased resilience and adaptability to changes and challenges in the educational landscape. Their proactive and positive approach allows them to navigate difficulties effectively, further safeguarding their mental health (Deroncele-Acosta et al., 2024). While existing literature supports a robust link between teacher engagement and positive mental health outcomes, there remains a gap in understanding this relationship within the specific context of Chinese College teachers. The unique pressures and demands faced by these educators, including heavy workloads, high expectations, and potential isolation, may necessitate a tailored approach to understanding and promoting engagement and mental well-being (Wang, 2024).

The reform of College Chinese teaching in Guangdong province has become imperative due to its current marginalization among students and teachers. There is a need for innovative teaching systems and enhancing the quality of teachers to improve the status and effectiveness of Chinese education in colleges (Chen, 2017). This reform can foster a more supportive and engaging environment for teachers, contributing to their mental well-being.

Moreover, research indicates significant differences in the curriculum of Chinese language teacher education programs between China and Australia, highlighting the urgent need for an internationalized curriculum to meet the demand for qualified teachers (Wang et al., 2013). Such international perspectives can inform the development of engagement strategies tailored to the specific needs of teachers in Guangdong province.

Chinese teachers have also been found to perceive lower levels of instructor immediacy compared to their American counterparts, which can impact their engagement and mental health (Myers et al., 1998). Addressing these perceptual gaps through targeted professional development and support can enhance teacher engagement and mental well-being.

### Mediating role of emotional intelligence (attention, clarity and repair) in the relationships between teachers’ engagement and mental health

Emotional intelligence (EI) is increasingly recognized as a critical factor in various aspects of life, including mental health and workplace well-being (Salovey et al., 2002; Sha et al., 2022). EI encompasses the ability to perceive, understand, use, and manage emotions in oneself and others (Seal et al., 2009). The components of EI relevant to this study are attention, clarity, and repair. Attention refers to the ability to focus on and be aware of emotions, clarity involves understanding and identifying emotions accurately, and repair pertains to regulating and managing emotions effectively.

Existing research suggests that EI plays a mediating role in the relationship between work engagement and mental health (Parris, 2021). Engaged teachers who possess high EI are better equipped to manage stress, build positive relationships, and navigate challenges, contributing to their mental well-being (Pyne, 2017). Attention allows them to recognize stressors early on, clarity enables them to understand the sources of stress and their emotional responses, and repair equips them with strategies to cope effectively (Sanchez-Pujalte et al., 2023).

Attention allows teachers to tune into their emotional state and the emotional climate of their classroom, facilitating early identification of potential stressors and enabling proactive interventions. Research by Seal et al. (2009) emphasizes the importance of understanding and identifying emotions accurately (clarity) to help teachers differentiate between various emotions and their underlying causes, allowing for more targeted and effective coping strategies (Valente and Lourenço, 2022). The ability to regulate emotions (repair) is crucial for managing stress and preventing burnout. Teachers with strong repair skills can employ healthy coping mechanisms such as mindfulness, relaxation techniques, and seeking support when needed (Menter, 2022).

Further, Lee and Chol (2018) found that EI plays a mediating role in the relationship between performance emotions and experiences, influencing both positive and negative emotions. This highlights the importance of EI in managing emotions effectively in high-stress environments. In a similar vein, Singh and Ryhal (2023) also demonstrated that EI significantly impacts teaching styles and the ability to handle classroom challenges, emphasizing the mediating role of EI in educational settings.

While previous studies have established the mediating role of EI in general work settings, its specific role in the context of Chinese college teachers remains under-explored. Given the unique stressors faced by these educators, understanding how EI influences their engagement and mental health is crucial for developing targeted interventions. This study aims to fill this gap by examining the mediating role of EI, specifically attention, clarity, and repair, in the relationship between teacher engagement and mental health among Chinese college teachers. This approach can provide insights into how to enhance teacher well-being through the development of emotional intelligence skills.

### Serial mediation between teacher engagement, emotional intelligence, and teacher–student relationships on teachers’ mental health

Emotional intelligence (EI) is a well-established factor in understanding how individuals manage their emotions and navigate interpersonal contexts. EI encompasses the ability to perceive, understand, use, and regulate emotions effectively. Among teachers, the components of EI—attention, clarity, and repair—play a critical role in shaping both internal coping mechanisms and external relational dynamics.

Teacher engagement, characterized by vigor, dedication, and absorption in one’s work, has been shown to positively influence EI. Engaged teachers are more emotionally attuned, motivated to reflect on their emotional states, and committed to emotional self-regulation in service of professional goals (Parris, 2021; Pyne, 2017). This heightened EI enables them not only to manage stress more effectively but also to engage in more responsive and empathetic interactions with students.

Indeed, emotional intelligence serves as a foundation for building high-quality teacher–student relationships. Teachers with strong emotional clarity and repair capacities are more likely to understand students’ needs, respond constructively to classroom challenges, and foster a trusting and respectful climate (Seal et al., 2009; Singh and Ryhal, 2023). Thus, EI acts as a relational enabler: it facilitates the social–emotional conditions necessary for the development of positive interpersonal bonds with students.

These teacher–student relationships, in turn, contribute significantly to teachers’ mental health. Positive interactions with students provide teachers with emotional affirmation, reduce feelings of professional isolation, and enhance their sense of purpose and fulfillment (Gan, 2021; Kemp et al., 2023). In this way, teacher–student relationships represent the relational pathway through which the intrapersonal benefits of EI translate into sustained psychological well-being.

Although the individual roles of engagement, EI, and teacher–student relationships have been explored in various contexts, few studies have examined their sequential interplay as a mechanism leading to mental health. A serial mediation model proposes that teacher engagement enhances emotional intelligence, which in turn fosters better teacher–student relationships, ultimately leading to improved mental health. This model recognizes that well-being is not the result of isolated factors but emerges from a cascade of interconnected psychological and relational processes.

In the specific context of Chinese college teachers—who often face high workloads and limited emotional support—this pathway may be especially relevant. By testing this serial mediation model, the present study aims to provide a theoretically grounded and empirically testable framework to inform evidence-based strategies for promoting teacher well-beings.

## Hypotheses

Based on the revised literature, the present study is aimed to test the following hypotheses:

### Direct effect

*H1*: Teacher engagement (X) has a significant positive effect on teachers’ mental health (Y) among Chinese college Teachers. As previous research supported, teacher engagement refers to the level of commitment, enthusiasm, and dedication that teachers bring to their work. Engaged teachers are typically more involved in their teaching activities, show higher levels of motivation, and are more resilient in the face of challenges.

### Indirect effect hypotheses (mediating variables)

The rationale for this set of hypotheses is that EI is a critical factor in understanding how individuals perceive, understand, and manage emotions. For teachers, high levels of EI can enhance their ability to cope with the emotional demands of their profession.

*H2*: The relationship between teacher engagement (X) and teachers’ mental health (Y) is mediated by EI attention (M1).

Literature showed that teachers who are more attentive to their emotions (EI attention) are better able to recognize and understand their emotional states. This heightened awareness can help them manage stress and emotional exhaustion, leading to better mental health outcomes.

*H3*: The relationship between teacher engagement (X) and teachers’ mental health (Y) is mediated by EI clarity (M2).

Clarity in understanding one’s emotions (EI clarity) allows teachers to interpret their emotional experiences accurately. This clarity can reduce confusion and emotional turmoil, contributing to improved mental health, supporting H3.

*H4*: The relationship between teacher engagement (X) and teachers’ mental health (Y) is mediated by EI repair (M3).

Research stated that the ability to repair negative emotions (EI repair) involves strategies to alleviate distress and enhance positive emotions. Teachers proficient in emotional repair are likely to recover more quickly from setbacks and maintain a positive emotional state, promoting better mental health.

*H5*: The relationship between teacher engagement (X) and teachers’ mental health (Y) is mediated by the quality of teacher–student relationships (M4).

Literature showed that the quality of relationships between teachers and students is a crucial factor in the educational environment. Positive teacher–student relationships can enhance teachers’ sense of fulfillment and job satisfaction, thereby improving their mental health. Strong, positive relationships with students can provide emotional support and a sense of accomplishment for teachers, reducing feelings of isolation and stress. This supportive environment can buffer against the negative effects of job-related stress, enhancing overall mental health.

### Serial mediation hypotheses

The serial mediation hypotheses (H6, H7, and H8) propose that teacher engagement impacts mental health through a two-step process: first, by enhancing specific aspects of emotional intelligence (attention, clarity, repair), and second, by improving the quality of teacher–student relationships. This sequential influence underscores the interconnectedness of emotional self-regulation and interpersonal dynamics in determining teachers’ mental health. Teachers who are engaged are more emotionally aware, clear, and resilient, which not only benefits their personal well-being but also translates into better relationships with students. These improved relationships, in turn, provide a supportive and fulfilling work environment that further enhances teachers’ mental health.

*H6*: Teacher engagement (X) affects teachers’ mental health (Y) through a serial mediation process involving EI attention (M1) and the quality of teacher–student relationships (M4).

As literature supported, Teachers with high engagement are more attentive to their emotions, allowing them to understand and manage their feelings effectively. This emotional attentiveness positively impacts the quality of their relationships with students, as teachers who are aware of their emotions are better equipped to create supportive and empathetic interactions with students. Finally, enhanced teacher–student relationships, in turn, provide emotional support and fulfillment, leading to better mental health outcomes.

*H7*: Teacher engagement (X) affects teachers’ mental health (Y) through a serial mediation process involving EI clarity (M2) and the quality of teacher–student relationships (M4).

As research supported, engaged teachers have clearer understanding of their emotional states, which allows for better emotional regulation and decision-making. Clear emotional understanding helps teachers foster more effective and meaningful relationships with students, as they can communicate and respond to students’ needs more appropriately. Positive teacher–student relationships resulting from emotional clarity contribute to reduced stress and increased job satisfaction, enhancing overall mental health.

*H8*: Teacher engagement (X) affects teachers’ mental health (Y) through a serial mediation process involving EI repair (M3) and the quality of teacher–student relationships (M4).

Teachers who are engaged and possess strong emotional repair skills can effectively manage and mitigate negative emotions, maintaining a positive emotional state. Effective emotional repair enables teachers to remain calm and composed, thereby improving their interactions and relationships with students. High-quality teacher–student relationships, bolstered by teachers’ ability to repair their own emotions, provide emotional resilience and support, leading to better mental health outcomes.

**Figure 1:**
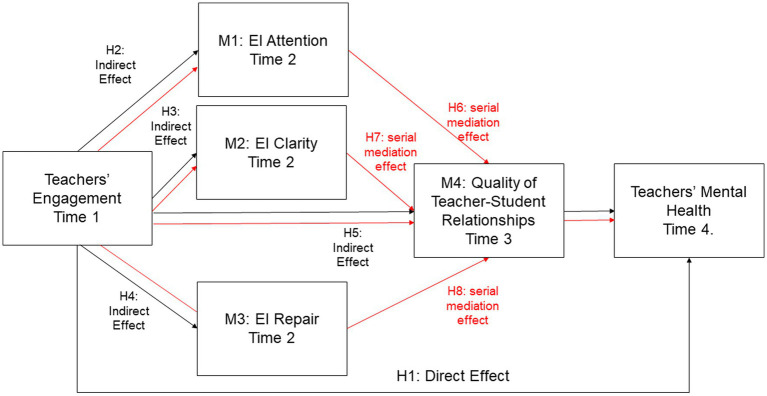
Hypotheses for the direct, indirect and serial mediation effects. Direct and indirect effects are displayed in black arrows; serial mediation effects are displayed in red arrows.

These hypotheses, that outline potential direct and indirect relationships among the variables of interest, considering both individual and combined mediating effects, are displayed in Figure 1.

## Method

### Participants

The final sample consisted of 1,264 Chinese college teachers from the Guangdong Province. Among these participants, there was a nearly even distribution of gender, with 621 male teachers, representing 49.1% of the sample, and 643 female teachers, representing 50.9% of the sample.

Regarding the type of college, the sample included teachers from both private and public institutions. Specifically, 574 teachers (15.4%) were affiliated with private colleges, whereas 690 teachers (84.6%) were from public colleges.

The educational levels of the participants varied. A significant portion of the teachers held advanced degrees, with 697 participants (55.1%) having a Master’s degree and 58 participants (4.6%) holding a PhD. Additionally, 482 teachers (38.1%) were college graduates.

The age range of the participants was from 23 to 60 years, with an average age of 42.04 years and a standard deviation of 9.822 years. The distribution of specialties among the participants aligns with the broader trends observed in Chinese higher education. Engineering stands out as the most prominent field (35%), with a substantial number of teachers specializing in sub-disciplines such as Civil, Mechanical, Electrical, and Computer Science & Engineering. Business and Economics (20%) follow as significant fields among the teachers. Specialties such as Finance, Accounting, and Marketing are particularly well-represented. Medicine holds a significant place among the teachers (15%), with specializations in Clinical Medicine, Nursing, and Pharmacy. Education (12%), with many teachers specializing in areas such as early childhood education, primary and secondary education, and special education. Law (8%), although a smaller field compared to others, as well as Agriculture and Forestry (4%) and Traditional Chinese medicine (6%).

### Procedure

Ethical approval for this study was obtained from the South China Normal University Ethical committee. The study adhered to the principles outlined in the Declaration of Helsinki and complied with local legislation. Given that there are more than 80 institutions of higher learning in Guangdong Province, all of them were invited to participate in the present research. To effectively disseminate research among higher education institutions in Guangdong province, social networks were used. Firstly, WeChat, known locally as 微信, stands out as the most influential platform in China. Secondly, Sina Weibo, akin to Twitter, which is a popular microblogging website in China. It facilitates rapid dissemination of information and is extensively used by the academic community. The survey was implemented using Qualtrics’ robust tools, which support various dissemination methods such as email, direct links, and social media sharing. This flexibility ensures wide reach and accessibility. At the beginning, were informed about the anonymity, voluntariness, compromise about data will not be send to any other parties, and all participants provided informed consent. To minimize potential common method biases (Podsakoff et al., 2003), data were collected over four distinct time periods within a span of 8 weeks. All participants provided informed consent. Participants created a private code to joint all the phases of the survey. At Time 1 (March of 2023), a total of 1,598 Chinese college teachers from the Guangdong province were initially surveyed to assess their engagement levels (X). Two weeks later, at Time 2, 1,454 participants (a reduction of 9% of participants) were surveyed again to measure their emotional intelligence (EI) in terms of attention (M1) and clarity (M2). Two weeks following Time 2, at Time 3, 1,348 participants (a reduction of 7,3% of participants) were surveyed for the third time to evaluate their emotional intelligence in terms of repair (M3) and the quality of their teacher–student relationships (M4). Two weeks after Time 3, at Time 4 (May, 2023), the participants’ mental health (Y) was assessed, with 1,264 participants (a reduction of 6,2% participants) completing the final survey. Throughout the four time periods, attrition occurred, resulting in a final sample of 1,264 participants who completed all four surveys.

### Measures

#### Teachers’ engagement

Teachers’ engagement was assessed using the Chinese version of the ultra-short Utrecht Work Engagement Scale (C-UWES-3), which has been validated for use in various Chinese contexts (Su et al., 2023). This version comprises three items (Items 2, 3, and 8) selected from the original nine-item UWES scale. These items were identified based on their highest factor loadings on the general factor of work engagement and are theoretically aligned with the core dimensions of vigor, dedication, and absorption. Specifically, vigor is measured by the item “At my job, I feel strong and vigorous”; dedication by “I am enthusiastic about my job”; and absorption by “I am immersed in my work.”

Participants responded to each item using a 5-point Likert-type scale ranging from 1 (never) to 5 (every day). It is important to note that this version differs slightly from the validated English version of the UWES-3 developed by Schaufeli et al. (2017), which includes Items 1, 3, and 8. The adaptation adopted in the Chinese version reflects culturally and psychometrically grounded decisions that enhance measurement validity in the local context. The C-UWES-3 also differs from other national versions, such as the Korean adaptation presented by Choi et al. (2020), reinforcing the importance of cultural specificity in the selection of engagement indicators.

#### Teachers’ emotional intelligence

In this study, we utilized the Trait Meta-Mood Scale (TMMS-24), a measure developed by Salovey et al. (2002) to assess perceived emotional intelligence. This scale consists of 24 items divided into three dimensions, each with eight items: (a) Emotional attention: This dimension evaluates the capacity to recognize and express emotions accurately (e.g., “I pay close attention to my feelings”). (b) Emotional clarity: This dimension measures the understanding of one’s emotional experiences (e.g., “I am clear about my feelings”). (c) Emotional repair: This dimension assesses the ability to regulate and manage emotions (e.g., “I try to have positive thoughts, even when I feel bad”).

Participants answered the items using a 5-point Likert scale, where 1 represents “strongly disagree” and 5 represents “strongly agree.” This questionnaire was employed by Li et al. (2002) with Chinese military students.

#### Quality of teacher–student relationships

The quality of teacher–student relationships was assessed using the Teacher–Student Relationship Inventory (TSRI), a new, concise self-report instrument designed to capture teachers’ perceptions of their interactions with students (Ang, 2005). Teachers evaluated the TSRI, which consists of 14 items, by indicating their level of agreement with each statement on a 5-point Likert scale. The response options ranged from 1 (almost never true) to 5 (almost always true). The TSRI included three aspects: Satisfaction (5 items): An example item is, “I would describe my relationship with this student as positive.” Instrumental Help (5 items): An example item is, “If this student needs help, he/she is likely to ask me for help.” Conflict (4 items): An example item is, “If this student is absent, I feel relieved.” Overall, the TSRI provides a reliable and valid global measure of the quality of teacher–student relationships, encompassing both positive and negative features.

#### Teachers’ mental health

The mental health assessment employed the five-scale Mental Health Test (MHT), a newly developed instrument designed to operationalize the Maintainable Positive Mental Health Theory (Zábó et al., 2022). This theoretical model emphasizes that mental health should not be assessed solely through the presence or absence of psychological symptoms, but rather as a sustainable, dynamic state of well-being. It highlights the importance of flexible adaptation, personal development, and the cultivation of supportive competencies and personality traits.

The MHT includes 18 items distributed across five dimensions of mental health, with three, three, five, three, and four items per dimension, respectively. These dimensions cover well-being, savoring, creative and executive efficiency, self-regulation, and resilience. Participants rate each item on a 5-point Likert scale ranging from 1 (does not agree at all) to 5 (agrees completely). The Well-being, Savoring, and Creative and Executive Efficiency subscales consist exclusively of positively worded items. The Self-regulation subscale includes only negatively worded items, while the Resilience subscale combines both positively and negatively phrased items. A final global score is computed by averaging the items within each dimension.

In addition to its solid theoretical grounding, the MHT has been recently applied in several empirical studies in China, demonstrating its cultural applicability and psychometric robustness. For instance, Chen et al. (2025) utilized the MHT to examine mental health problems among boarding adolescents in rural China, while Guan et al. (2025) employed the scale to assess the association between commuting time, academic performance, and mental health. Likewise, Yu et al. (2025) and Ding et al. (2025) incorporated the MHT in studies on myopia and psychological well-being among schoolchildren, and Dai et al. (2025) used the instrument in an intervention-based mental health evaluation. These studies confirm the instrument’s utility across diverse educational and health-related contexts in China, supporting its validity for the present research on college teachers.

Overall, the MHT offers a validated and contextually sensitive tool for capturing the multidimensional nature of mental health, aligned with both contemporary theoretical perspectives and empirical applications in Chinese populations.

### Data treatment

Descriptive statistics were calculated using SPSS 29.01 to summarize the basic features of the data. This included calculating the mean, standard deviation, minimum, and maximum values for continuous variables such as age. For categorical variables like gender, type of college, and educational level, frequencies and percentages were computed. A chain mediation model using the PROCES 4.2. macros developed by Hayes (2022) was employed to investigate the relationships between teachers’ engagement (measured at time 1) and teachers’ mental health (measured at time 4), with emotional intelligence components (EI attention, EI clarity, EI repair, all measured at time 2) and the quality of teacher–student relationships (measured at time 3) as mediators. Specifically, Model 80 was used. To assess the significance of the mediation effects, the bootstrapping method was employed. Bootstrapping is a non-parametric resampling technique that involves repeatedly sampling from the data with replacement to create many simulated samples. This method is particularly useful for estimating the sampling distribution of a statistic (e.g., a mediation effect) and for constructing confidence intervals. The bootstrapped confidence intervals provide a range of values within which the true parameter is likely to fall. In this study, the cut-off values for the upper limit confidence interval (ULCI) and the lower limit confidence interval (LLCI) were used to determine the significance of the mediation effects. If the confidence interval for a mediation effect does not include zero, the effect is considered statistically significant. The Model 80 included: X (Independent Variable): Teachers’ Engagement assessed at Time 1; (M1): EI Attention assessed at Time 2, M2: EI Clarity assessed at Time 2, M3: EI Repair assessed at Time 2, M4: Quality of Teacher–Student Relationships assessed at Time 3, and Y (Dependent Variable): Teachers’ Mental Health assessed at Time 4.

## Results

### Descriptive statistics and correlation matrix

The descriptive statistics for the variables are presented in Table 4. The variables include Teachers’ Engagement (X), EI Attention (M1), EI Clarity (M2), EI Repair (M3), Quality of Teacher–Student Relationships (M4), and Teachers’ Mental Health (Y). The table provides the means and standard deviations for each variable, demonstrating the central tendency and dispersion of the data.

The correlation matrix is in Table 1, reveals significant relationships between several variables. Teachers’ Engagement (X) is positively correlated with all other variables, indicating that higher engagement is associated with higher levels of EI Attention (M1), EI Clarity (M2), EI Repair (M3), Quality of Teacher–Student Relationships (M4), and Teachers’ Mental Health (Y). Notably, the strongest correlations are observed between Teachers’ Engagement (X) and Teachers’ Mental Health (Y), as well as between Teachers’ Engagement (X) and Quality of Teacher–Student Relationships (M4).

**Table 1:** Descriptive statistics and correlation matrix.

Variable	*M*	SD	1	2	3	4	5	6
1. Teachers’ engagement (X)	3.57	0.99	*0.78*					
2. EI attention (M1)	3.42	0.78	0.10**	*0.82*				
3. EI clarity (M2)	3.73	0.64	0.26**	0.16**	*0.84*			
4. EI repair (M3)	3.79	0.69	0.31**	0.09**	0.40**	*0.72*		
5. Quality of teacher–student relationships (M4)	3.89	0.83	0.55**	0.05	0.26**	0.22**	*0.80*	
6. Teachers’ mental health (Y)	4.86	1.06	0.76**	0.14**	0.29**	0.41**	0.57**	*0.85*

*N* = 1,264. **p < 0.01. Reliability indices are the values in the diagonal in italics.

EI Attention (M1) is positively correlated with EI Clarity (M2) and EI Repair (M3), suggesting that teachers who are attentive to their emotions also tend to have clearer understanding and better repair of their emotions. EI Clarity (M2) and EI Repair (M3) are also positively correlated, further supporting this relationship. Quality of Teacher–Student Relationships (M4) is positively correlated with all aspects of Emotional Intelligence (M1, M2, M3) and Teachers’ Mental Health (Y), highlighting the role of positive interactions in the educational environment.

The correlation between EI Attention (M1) and Quality of Teacher–Student Relationships (M4) is not significant, which may suggest that mere attentiveness to emotions without clarity or repair does not necessarily translate into better relationships with students. The significant positive correlations between all other variables suggest that higher levels of engagement and emotional intelligence are associated with better teacher–student relationships and improved mental health outcomes.

The descriptive statistics and correlation matrix highlight significant relationships between teacher engagement, emotional intelligence facets, quality of teacher–student relationships, and teachers’ mental health. Teacher engagement shows strong positive correlations with both quality of teacher–student relationships and teachers’ mental health, indicating that higher engagement is crucial for well-being and positive relational dynamics. Notably, no statistically significant correlation was found between EI attention and the quality of teacher–student relationships (*r* = 0.05, ns). This lack of association suggests that while emotional awareness may support individual emotional processes, it does not necessarily translate into stronger relational bonds with students, at least within this sample. Emotional intelligence, particularly EI Clarity and EI Repair, is also positively associated with better mental health and teacher–student relationships, emphasizing the importance of emotional competencies in the educational context.

### Direct effect of teacher engagement on mental health

The direct effect of teacher engagement on teachers’ mental health was significant. The results indicate that higher levels of teacher engagement are associated with better mental health among Chinese College teachers. This finding is supported by the statistical values presented in Table 2.

**Table 2:** Direct effect of teacher engagement on teachers’ mental health.

Predictor	*B*	SE	*t*	*p*	LLCI	ULCI	*β*
Constant	0.245	0.146	1.678	0.094	−0.042	0.532	
Teacher engagement	0.621	0.022	27.848	0.000	0.577	0.665	0.579

B = Unstandardized Regression Coefficient; SE = Standard Error; LLCI = Lower Limit of the 95% Confidence Interval; ULCI = Upper Limit of the 95% Confidence Interval; β = Standardized Regression Coefficient. The results show a statistically significant direct effect of teacher engagement on teachers’ mental health (*p* < 0.001). Confidence intervals are based on 5,000 bootstrap samples.

### Indirect effects of teacher engagement on mental health

The mediation analysis revealed several significant indirect effects of teacher engagement on teachers’ mental health. Firstly, the mediation effect through EI attention was significant, indicating that increased teacher engagement enhances EI attention, which in turn improves mental health. Secondly, the mediation effect through EI repair was also significant, suggesting that teacher engagement boosts the ability to repair emotions, leading to better mental health outcomes. Conversely, the mediation effect through EI clarity was not significant.

Additionally, the quality of the teacher–student relationship significantly mediated the relationship between teacher engagement and mental health, underscoring the importance of positive interpersonal interactions in the educational environment. These mediation effects are detailed in Table 3.

**Table 3:** Indirect effects of teacher engagement on teachers’ mental health.

Indirect path	Effect	BootSE	BootLLCI	BootULCI	*β*
Total Indirect Effect	0.192	0.020	0.154	0.231	0.179
Teacher engagement → EI attention → Teacher mental health	0.006	0.003	0.002	0.012	0.006
Teacher engagement → EI clarity → Teacher mental health	0.002	0.006	−0.008	0.014	0.002
Teacher engagement → EI repair → Teacher mental health	0.059	0.008	0.043	0.074	0.055
Teacher engagement → Quality of teacher–student relationships → Teacher mental health	0.118	0.016	0.086	0.150	0.110

BootSE = Bootstrapped Standard Error; BootLLCI = Lower Limit of the 95% Bootstrapped Confidence Interval; BootULCI = Upper Limit of the 95% Bootstrapped Confidence Interval; β = Standardized Indirect Effect. Confidence intervals were calculated using 5,000 bootstrap samples. Indirect effects are considered statistically significant when the 95% confidence interval does not include zero.

### Serial mediation effects

The serial mediation analysis examined pathways involving emotional intelligence (EI) facets and the quality of teacher–student relationships as sequential mediators. Three serial indirect paths were tested.

Firstly, the serial path from teacher engagement through EI attention and teacher–student relationship quality to mental health was negative and not statistically significant (*β* = −0.006; 95% CI: −0.021, 0.005). This suggests that although EI attention alone may positively influence relational sensitivity, when combined sequentially with relationship quality, the cumulative effect does not contribute significantly to improved mental health. The negative coefficient may indicate a complex interplay in which increased emotional awareness could amplify sensitivity to relational tensions, potentially diluting the psychological benefits.

Secondly, the serial path through EI clarity and teacher–student relationship quality was statistically significant (*β* = 0.007; 95% CI: 0.004, 0.011). This finding supports the hypothesis that teacher engagement enhances emotional clarity, which in turn strengthens relational dynamics, ultimately leading to better mental health. This pathway underscores the importance of clearly understanding and interpreting emotional experiences in fostering constructive interpersonal connections that support well-being.

Thirdly, the serial path through EI repair and teacher–student relationship quality was not significant (*β* = 0.002; 95% CI: −0.003, 0.004). Although EI repair alone had a significant indirect effect in the simple mediation model, its sequential influence through relationship quality did not reach statistical significance. This may suggest that while emotional regulation is beneficial for individual coping, it may not substantially contribute to relational processes that influence mental health outcomes in a serial framework.

**Figure 2:**
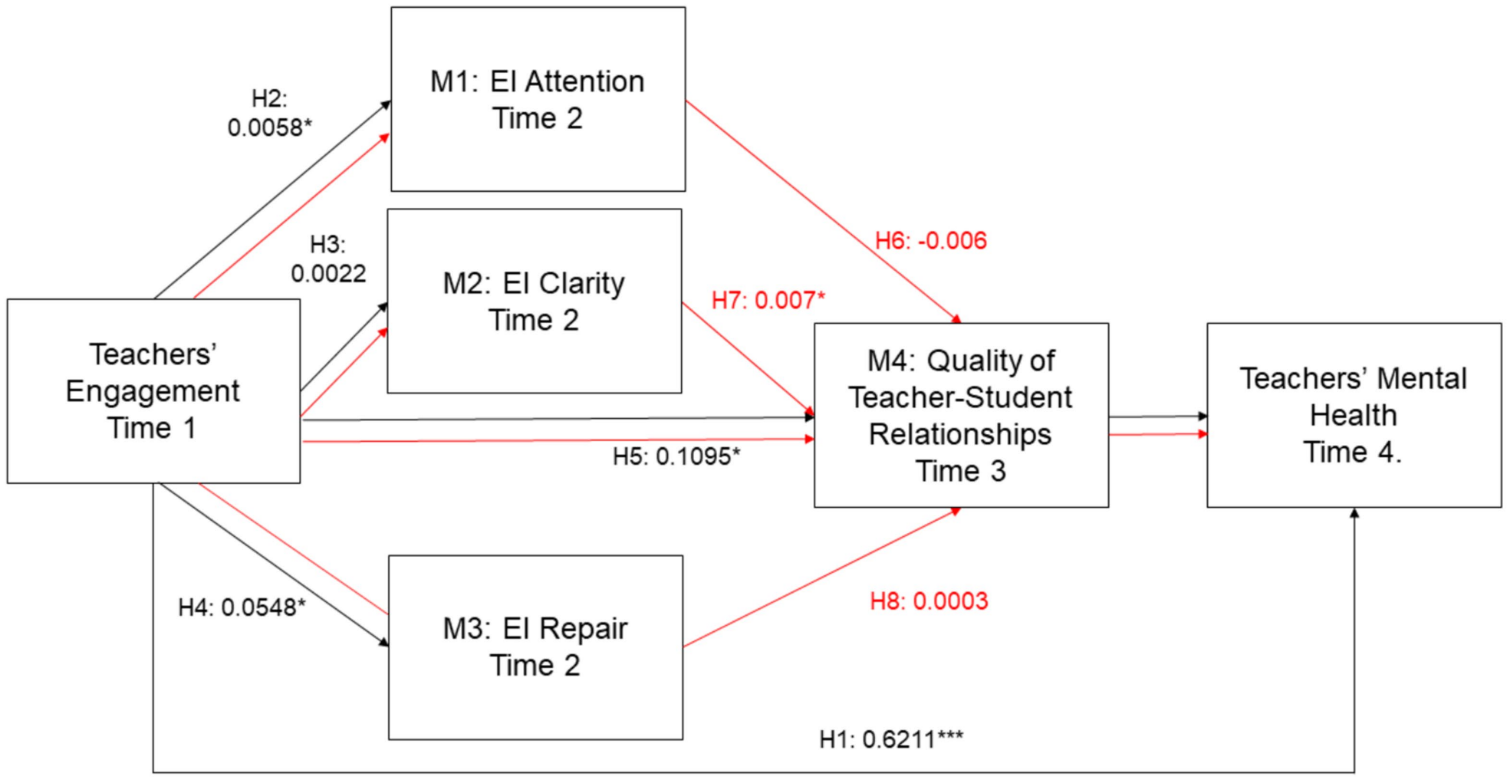
Standardized estimates for the direct, indirect and serial mediation effects.

Together, these findings indicate that among the tested pathways, only the sequence involving EI clarity and relationship quality operates as a significant serial mediator. All serial mediation results are summarized in Table 4, and the standardized estimates are illustrated in Figure 2.

**Table 4:** Serial mediation effects of teacher engagement on teachers’ mental health.

Serial path	BootSE	BootLLCI	BootULCI	*β*
Teacher engagement → EI attention → Quality of teacher–student relationships → Teacher mental health	0.006	−0.021	0.005	−0.006
Teacher engagement → EI clarity → Quality of teacher–student relationships → Teacher mental health	0.002	0.004	0.011	0.007
Teacher engagement → EI repair → Quality of teacher–student relationships → Teacher mental health	0.002	−0.003	0.004	0.002

BootSE = Bootstrapped Standard Error; BootLLCI = Lower Limit of the 95% Bootstrapped Confidence Interval; BootULCI = Upper Limit of the 95% Bootstrapped Confidence Interval; β = Standardized Indirect Effect. Confidence intervals were calculated using 5,000 bootstrap samples. An indirect effect is considered statistically significant when the 95% confidence interval does not include zero.

## Discussion

The aim of the present study was to test a chain mediation model of Emotional intelligence dimension (attention, clarity and repair) and Quality of teacher–student relation into the relationships between Teachers’ engagement and Teachers’ Mental Health.

### Direct effect

The analysis revealed a statistically significant direct effect of teacher engagement on teachers’ mental health, suggesting that, within this sample, higher levels of engagement are generally associated with better mental health outcomes. This aligns with previous research indicating that engaged teachers tend to experience higher job satisfaction, reduced stress, and lower levels of burnout, all of which contribute to overall psychological well-being (Bakker and Demerouti, 2008; Schaufeli and Bakker, 2004).

However, the strength and direction of this association may vary across contexts. McMullen and Shepherd (2016), for instance, have pointed out that high engagement can sometimes intensify work demands, potentially resulting in increased stress and reduced well-being. Such findings caution against assuming a uniformly positive link between engagement and mental health. Factors such as institutional environment, workload, support systems, and individual coping mechanisms may play a moderating role in this relationship.

Taken together, the current results support a direct positive effect, but they also highlight the need for further investigation into the conditions under which engagement contributes to or detracts from mental health in educational settings.

### Indirect effect

The mediation analysis revealed several significant indirect effects of teacher engagement on teachers’ mental health, mediated by facets of emotional intelligence.

The mediation effect through EI attention was significant, suggesting that increased teacher engagement enhances EI attention, which in turn improves mental health. This finding aligns with the theory that being more attuned to one’s emotions helps manage stress and emotional challenges, leading to better mental health outcomes (Goleman, 1995). Conversely, Felton et al. (2015) argue that heightened emotional attention might sometimes lead to over-sensitivity to emotional cues, potentially exacerbating stress if not managed properly. Thus, while the overall effect is positive, individual differences in handling increased emotional attention could vary.

The mediation effect through EI repair was also significant, indicating that teacher engagement boosts the ability to repair emotions, leading to better mental health outcomes. This supports the notion that effective emotional regulation is crucial for maintaining mental health, particularly in high-stress professions like teaching (Mayer et al., 2004; Mayer et al., 2001). However, it is important to note that the ability to repair emotions might also depend on external factors such as the availability of support networks and institutional resources, which were not controlled for in this study.

Conversely, the mediation effect through EI clarity was not significant. This result suggests that while understanding and identifying emotions accurately (clarity) is valuable, it does not independently mediate the relationship between teacher engagement and mental health as strongly as EI attention and repair. This finding raises questions about the distinct roles of different EI components and suggests that clarity alone may not be sufficient to influence mental health without being coupled with other emotional competencies.

### Serial mediation

The serial mediation analysis examined pathways involving emotional intelligence (EI) facets and the quality of teacher–student relationships. The indirect effect through EI attention and teacher–student relationship quality was negative and not statistically significant. While EI attention alone showed a significant simple mediation effect, this result suggests that when combined in sequence with relational factors, the outcome does not translate into improved mental health.

One possible explanation is that heightened emotional attention, while valuable for internal awareness, may increase teachers’ sensitivity to subtle relational tensions or emotional dynamics in the classroom. Rather than fostering positive interactions, this heightened awareness may sometimes magnify perceived interpersonal stress, leading to disengagement or emotional strain (Felton et al., 2015). In contexts where emotional demands are already high, increased emotional monitoring may be cognitively taxing or emotionally overwhelming, particularly if not accompanied by clarity or regulation skills.

Moreover, the absence of a positive serial effect suggests that emotional attention alone may not be sufficient to improve relationship quality—and thus, mental health—unless it is supported by other emotional competencies such as clarity or repair. Future research could investigate whether combinations of EI skills (e.g., attention + clarity) act in a complementary way to promote more adaptive relational outcomes. These findings highlight that emotional awareness, although beneficial in moderation, can have neutral or even adverse implications if not integrated within a broader set of emotional regulation capacities.

The present results are in line with recent research showing that teacher–student relationship plays a significant moderator role in the relationship between emotional intelligence and academic performance, mediated by flourishing (Chamizo-Nieto et al., 2021). Despite testing different hypotheses, their findings suggest that both emotional intelligence and teacher–student relationship quality are positively related to flourishing, and that their interaction may counterbalance their independent effects on this personal well-being indicator. This complexity underscores the need for a nuanced understanding of how emotional awareness impacts relational and mental health outcomes.

In contrast, the indirect effect through EI clarity and teacher–student relationship quality was statistically significant, indicating that teacher engagement enhances EI clarity, which improves the quality of teacher–student relationships, ultimately leading to better mental health. This finding highlights the importance of clear emotional understanding in fostering positive interpersonal interactions, which in turn support mental health. It suggests that clarity in emotional perception can enhance the quality of relationships, contributing to a more supportive and fulfilling work environment.

This finding may indicate that EI clarity primarily facilitates outcomes through its effect on interpersonal processes rather than through intrapersonal regulation alone. While understanding and labeling one’s emotions does not appear to directly influence mental health in isolation, it may serve as a critical prerequisite for developing constructive social interactions. Teachers who are better able to interpret their own emotional experiences may be more effective in expressing themselves clearly, interpreting student cues accurately, and responding empathically—thus enhancing the quality of teacher–student relationships. These improved relationships, in turn, offer emotional reinforcement and reduce isolation, contributing positively to mental health. In this sense, EI clarity may function less as a self-regulatory mechanism and more as a social–emotional competency that enables stronger relational bonds, which ultimately support well-being.

In a related vein, the study by Chen et al. (2021) demonstrated that a serial mediator model showed that proactive personality was sequentially related to academic engagement through teacher–student relationships and academic self-efficacy. This supports the idea of chain mediation, where teacher–student relationships play a dual role: they are influenced by other predictors and simultaneously act as a bridge to the final outcomes. While most empirical research has focused on the impact of teacher–student relationships on student outcomes (Zheng, 2022), the present study highlights that consequences for teachers should also be considered.

The indirect effect through EI repair and teacher–student relationship quality was not significant. This result indicates that while EI repair is beneficial for individual emotional regulation, its impact on mental health through the pathway of teacher–student relationships is limited. It suggests that other factors may influence the dynamics of teacher–student relationships more significantly than the ability to repair emotions. Relevant studies have also tested a reverse model, in which teachers’ emotional intelligence predicted students’ objectively assessed academic achievement through teachers’ work engagement (Wang, 2022). Although the findings of the present study—based on a four-wave panel design—partially support the proposed hypotheses, more rigorous longitudinal studies are needed to provide stronger evidence for the directionality of causation.

In summary, the results of this study demonstrate that teacher engagement directly improves teachers’ mental health, with significant indirect effects mediated by emotional intelligence facets such as EI attention and EI repair, as well as the quality of teacher–student relationships. Notably, the serial mediation through EI clarity and teacher–student relationships further highlights the importance of clear emotional understanding and positive interpersonal interactions in enhancing mental health. The negative, though non-significant, indirect path through EI attention and teacher–student relationship quality suggests a potential complexity in how emotional awareness and relational dynamics interact. This may indicate that heightened EI attention could sometimes lead to increased sensitivity to relational issues that do not straightforwardly translate into mental health benefits. Further research is needed to explore these complex interactions and to identify strategies that can optimize the benefits of teacher engagement on mental health through improved emotional intelligence and teacher–student relationships.

### Limitations of the present study

While this study provides valuable insights into the relationships between teachers’ engagement, emotional intelligence, teacher–student relationships, and mental health, several limitations should be acknowledged.

First, the study was conducted with a regionally specific sample of Chinese college teachers from Guangdong Province. This regional focus may limit the generalizability of the findings to other provinces or cultural contexts. In addition, the voluntary nature of participation and the multi-wave data collection process may have introduced attrition bias, although no formal analysis was conducted to assess this potential effect.

Second, the study did not incorporate statistical control variables (e.g., gender, age, seniority, teaching discipline), which could influence both engagement and mental health. Future research should include control measures to better isolate the unique contributions of emotional intelligence and relational factors.

Third, all data were collected through self-report instruments, which can introduce common method bias and social desirability effects. Although procedural remedies were applied—such as multi-wave data collection—the exclusive reliance on self-reports remains a methodological limitation.

Fourth, despite the use of a four-wave panel design, the observational nature of the study precludes strong causal inferences. More rigorous longitudinal or experimental designs are needed to establish directionality among the variables studied.

Fifth, while the instruments employed are validated and reliable, they may not fully capture the nuanced and multidimensional nature of constructs such as emotional intelligence or mental health. Combining quantitative approaches with qualitative methods could enhance the depth and contextual relevance of future findings.

Sixth, the study focused solely on teachers’ self-perceptions, without incorporating perspectives from students, administrators, or peers. Including multiple viewpoints would provide a more holistic understanding of the relational dynamics that affect teacher well-being.

Finally, although the study acknowledges the collectivist cultural orientation of the Chinese educational context, the interaction between culture and the psychological constructs measured warrants further investigation. Future studies should explore how cultural norms, values, and institutional settings influence the processes identified here and their generalizability to other cultures.

### Suggestions for career counselors and human resource management at colleges

Based on the findings and discussion of this study, several practical recommendations can be made for career counselors and HRM professionals in college settings to enhance teacher engagement and, consequently, improve teachers’ mental health.

Firstly, career counselors and HRM professionals should prioritize creating a supportive and collaborative work environment. Recent qualitative studies, including informal interviews with medical professionals, organizational leaders in the public sector, teachers, and HR professionals, have highlighted significant challenges to employees’ mental health in the Chinese context (Cooke and Xu, 2024). Encouraging a culture of mutual support and open communication can help reduce feelings of isolation and stress among teachers. This can be achieved through regular team-building activities, peer support groups, and mentorship programs where experienced teachers support new or struggling colleagues. Such initiatives foster a sense of community and belonging, which are essential for maintaining positive mental health.

Secondly, given the significant role of emotional intelligence in mediating the relationship between teacher engagement and mental health, colleges should offer EI training programs. These programs can focus on developing skills in emotional attention, clarity, and repair. Workshops, seminars, and ongoing training sessions can help teachers become more aware of their emotions, understand them better, and learn effective strategies for emotional regulation.

Specifically, considering the features of collectivist culture, Chinese teachers may face unique challenges. As Chiu and Kosinski (1995) noted, Chinese workers tend to interpret and handle work-related stress differently from Westerners, even though they have been exposed to Western business practices. Similar to Asian Americans, they often find themselves caught between their collectivistic traditions and the increasingly competitive individualistic academic demands.

Enhancing the emotional attention, clarity, and repair components can equip teachers with the tools needed to manage stress effectively and maintain their well-being.

Improving the quality of teacher–student relationships is crucial for the mental health of teachers. HRM can facilitate this by organizing professional development sessions that focus on building positive relationships with students. Training in effective communication, conflict resolution, and empathy can help teachers create a more supportive and engaging classroom environment. Additionally, implementing a team of university counselors can help students cope with academic demands and navigate teacher–student relationships in higher education (Gao, 2024). Strong teacher–student relationships not only enhance students’ learning experiences but also provide emotional support to teachers, contributing to their mental health.

Additionally, career counselors and HRM professionals should ensure that teachers have easy access to mental health resources. This includes providing information about available counseling services, stress management workshops, and other mental health support programs. Regular mental health check-ins and promoting a culture where seeking help is normalized can also be beneficial. A person-centered approach could be useful in helping teachers develop their career adaptability and professional identity, which in turn could positively impact their mental health (Yan et al., 2024). Teachers should feel that their mental health is a priority and that resources are available to support them when needed.

Finally, encouraging professional development and growth is essential. Career counselors and HRM professionals should support teachers in pursuing further education, attending workshops, and participating in professional development opportunities. Engaged teachers who continuously develop their skills and knowledge feel more competent and confident, which positively impacts their mental health. Providing opportunities for career advancement and recognizing teachers’ achievements can also enhance their engagement and job satisfaction.

By implementing these strategies, career counselors and HRM professionals can create a work environment that supports teacher engagement and mental health, ultimately leading to a more effective and satisfied teaching workforce.

## Conclusion

The present study aimed to examine a chain mediation model involving dimensions of emotional intelligence (EI) and the quality of teacher–student relationships in the relationship between teachers’ engagement and their mental health. The findings revealed a significant direct effect of teacher engagement on mental health, suggesting that higher levels of engagement are associated with better mental health outcomes among Chinese college teachers, supporting existing literature indicating that engaged teachers experience higher job satisfaction, reduced stress, and lower burnout rates. However, the complexity of this relationship warrants further investigation, considering individual and contextual factors.

Mediation analyses showed significant indirect effects through specific EI components, with EI attention and EI repair significantly mediating the relationship between teacher engagement and mental health, aligning with theories suggesting that emotional attunement and effective emotional regulation are crucial for mental health, particularly in high-stress professions like teaching. However, the mediation effect through EI clarity was not significant, indicating that while emotional understanding is valuable, it does not independently mediate this relationship as strongly as EI attention and repair. The serial mediation analysis highlighted that EI clarity, when coupled with quality teacher–student relationships, significantly mediated the relationship between teacher engagement and mental health, underscoring the importance of clear emotional understanding in fostering positive interpersonal interactions, which, in turn, support mental health. Conversely, the pathways involving EI attention and EI repair, combined with teacher–student relationships, were not significant, suggesting a nuanced interaction where heightened emotional awareness might sometimes lead to increased sensitivity to relational dynamics, which may not always positively impact mental health.

The study highlights the critical role of fostering teacher engagement and developing emotional intelligence to enhance teachers’ mental health, suggesting that professional development programs focusing on EI training and strategies to improve teacher–student relationships could be beneficial. Additionally, understanding the complex interactions between emotional intelligence components and relational dynamics can inform more targeted interventions. Given the limitations of the study, including its regional focus and reliance on self-reported data, future research should aim to replicate these findings in different cultural contexts and with more rigorous longitudinal designs. Exploring the impact of external support systems and institutional resources on these relationships could also provide deeper insights. In summary, the study underscores the importance of teacher engagement and emotional intelligence in promoting mental health among teachers, with a particular emphasis on the roles of emotional clarity and positive teacher–student relationships, offering valuable guidance for educational institutions aiming to support the well-being of their teachers through targeted interventions and supportive environments.

## Data Availability

The raw data supporting the conclusions of this article will be made available by the authors, without undue reservation.
